# Intensity of perinatal care, extreme prematurity and sensorimotor outcome at 2 years corrected age: evidence from the EPIPAGE-2 cohort study

**DOI:** 10.1186/s12916-018-1206-4

**Published:** 2018-12-05

**Authors:** Andrei S. Morgan, Laurence Foix L’Helias, Caroline Diguisto, Laetitia Marchand-Martin, Monique Kaminski, Babak Khoshnood, Jennifer Zeitlin, Gérard Bréart, Xavier Durrmeyer, François Goffinet, Pierre-Yves Ancel

**Affiliations:** 1 INSERM UMR 1153, Obstetrical, Perinatal and Pediatric Epidemiology Research Team (EPOPé), Centre for Epidemiology and Statistics Sorbonne Paris Cité, DHU Risks in Pregnancy, Paris Descartes University, Hôpital Tenon, Rue de la Chine, Paris, 75020 France; 2 Institute for Womens’ Health, UCL, 74 Huntley Street, London, WC1E 6AU UK; 3 SAMU 93 - SMUR Pédiatrique, CHI André Gregoire, Groupe Hospitalier Universitaire Paris Seine-Saint-Denis, Assistance Publique des Hôpitaux de Paris, Montreuil, France; 4 UPMC Université Paris 6, Sorbonne Universités, Paris, France; 5 Service de Néonatologie, Hôpital Armand Trousseau, Assistance Publique des Hôpitaux de Paris, Paris, France; 6 Maternité Olympe de Gouges, Centre Hospitalier Regional Universitaire Tours, Tours, France; 7 Université François Rabelais, Tours, France; 8 Service de Médecine Néonatale, Centre Hospitalier Intercommunal de Creteil, Clinical Research Center CHI Créteil, Créteil, France; 9 Maternité Port-Royal, University Paris-Descartes, Hôpitaux Universitaires Paris Centre, Assistance Publique des Hôpitaux de Paris, Paris, France; 10 URC CIC P1419, DHU Risk in Pregnancy, Cochin Hotel Dieu, Assistance Publique des Hôpitaux de Paris, Paris, France

**Keywords:** Extreme prematurity, Newborn, Perinatal intensity, Activity, Obstetric, Neonatal, Epidemiology, Cohort study, Vital status, Neonate

## Abstract

**Background:**

Emerging evidence suggests intensity of perinatal care influences survival for extremely preterm babies. We evaluated the effect of differences in perinatal care intensity between centres on sensorimotor morbidity at 2 years of age. We hypothesised that hospitals with a higher intensity of perinatal care would have improved survival without increased disability.

**Methods:**

Foetuses alive at maternal admission to a level 3 hospital in France in 2011, subsequently delivered between 22 and 26 weeks gestational age (GA) and included in the EPIPAGE-2 national prospective observational cohort study formed the baseline population. Level of intensity of perinatal care was assigned according to hospital of birth, categorised into three groups using ‘perinatal intensity’ ratios (ratio of 24–25 weeks GA babies admitted to neonatal intensive care to foetuses of the same GA alive at maternal admission to hospital). Multiple imputation was used to account for missing data; hierarchical logistic regression accounting for births nested within centres was then performed.

**Results:**

One thousand one hundred twelve foetuses were included; 473 survived to 2 years of age (126 of 358 in low-intensity, 140 of 380 in medium-intensity and 207 of 374 in high-intensity hospitals). There were no differences in disability (adjusted odds ratios 0.93 (95% CI 0.28 to 3.04) and 1.04 (95% CI 0.34 to 3.14) in medium- and high- compared to low-intensity hospitals, respectively). Compared to low-intensity hospitals, survival without sensorimotor disability was increased in the population of foetuses alive at maternal admission to hospital and in live-born babies, but there were no differences when considering only babies admitted to NICU or survivors.

**Conclusions:**

No difference in sensorimotor outcome for survivors of extremely preterm birth at 2 years of age was found according to the intensity of perinatal care provision. Active management of periviable births was associated with increased survival without sensorimotor disability.

**Electronic supplementary material:**

The online version of this article (10.1186/s12916-018-1206-4) contains supplementary material, which is available to authorized users.

## Background

Extremely preterm infants, defined as those born at a gestational age (GA) between 22 and 26 weeks, represent 0.2–0.3% of all births [1, 2] but remain at high risk of mortality, neonatal morbidity and later developmental disorders [3, 4]. Evidence-based management has been shown to improve outcomes of these babies. Strategies include medical treatments like administration of antenatal steroids, appropriate early respiratory management and prevention of neonatal hypothermia following delivery, as well as organisational changes to promote delivery in a unit with appropriate neonatal facilities [5].

It is recognised that decision-making is important in determining outcome. Case selection occurs in the delivery room [6], and there is international variability in management of (threatened) extremely preterm deliveries. For example, national guidelines in the UK, north American and Scandinavian countries are more likely to recommend proactive or individualised care; other countries recommend comfort care or have no recommendations [7, 8]. A study looking at hospital-level aggregates of treatments provided to a population of live-born babies demonstrated improvements in survival and survival without severe morbidity in babies of 22 and 23 weeks receiving ‘active’ care following delivery when compared with the entire population of babies born at those gestations [9].

Case selection also occurs antenatally [10]. It has therefore been proposed that a ‘foetuses-at-risk’ approach should be adopted to minimise potential bias [11–13]. Evidence regarding the influence of intensity of perinatal care—that is, the degree to which women who deliver extremely preterm and their offspring receive ‘active’ management—on survival and morbidity outcomes is limited: only one study has examined this question. Indices of obstetric and neonatal treatments were created at a regional level using data from the Swedish EXPRESS cohort; survival and morbidity-free survival at 2.5 years of age were better in the more active regions [14]. Yet active management represents more than just treatment: as well as easily quantifiable measures (e.g. the proportions of women receiving antenatal steroids or babies intubated at delivery), there are underlying intentions to treat. These are difficult to assess and not necessarily accounted for by measuring treatments provided. Furthermore, indices based on treatments are difficult to transpose between studies due to differences in which data are collected and how.

The French EPIPAGE-2 national cohort collected data on all extremely preterm births in 2011 and will follow up survivors until 12 years of age [15]. French guidelines advise that perinatal management is based on assessment of the individual’s situation at 24 and 25 weeks gestation [16, 17]. Below 24 weeks, palliative care is recommended [17], whereas at 26 weeks, most babies are admitted to intensive care [2]. EPIPAGE-2 demonstrated national rates of survival to discharge of 31% and 60% of babies born at 24 and 25 weeks GA, respectively; 79% of liveborn babies were born in a level 3 centre [2, 18]. Results at 2 years corrected age were recently published [4]. These were based on physician assessment of sensorimotor status, as well as parentally reported Ages and Stages Questionnaire which is a screening tool for child neurodevelopmental status, including both intellectual/cognitive as well as motor abilities [19].

We decided to investigate the effects of variation in the intensity of active perinatal care at a hospital level using the EPIPAGE-2 cohort. Our objective was to evaluate this with a simple metric based upon readily available vital status data relating to births at 24 and 25 weeks of gestation and to assess the impact on objective outcomes available at 2 years of age—namely, physician-assessed sensorimotor disability and survival. We hypothesised that hospitals with a higher level of intensity of perinatal care would not have increased levels of sensorimotor morbidity compared with hospitals of lower intensity levels.

## Methods

### Study population

Methods of case identification, data capture and other design aspects for the EPIPAGE-2 cohort have been described previously [15]. All births in France between 22 and 26 completed weeks of gestation (i.e. 26 weeks and 6 days or fewer) occurring over an 8-month period in 2011 were included [15]. For this study, the population was restricted to mothers where the foetus was alive at admission to hospital and at either the start of monitoring of the labour or when it was decided to perform caesarean section. Foetuses with congenital lethal malformations and terminations of pregnancy for congenital anomalies were excluded. All births at 22 to 26 weeks gestation occurring in a level 3 hospital [20] with at least one delivery at 24 or 25 weeks gestation were included.

### Outcomes

The primary outcome was sensorimotor deficiency at 2 years of age among survivors. This consisted of the adverse findings of sensory disability (blindness in one or both eyes and/or unilateral or bilateral deafness) or cerebral palsy (assessed by the attending physician and defined according to the diagnostic criteria of the Surveillance of Cerebral Palsy in Europe (SCPE) network with independent review of ambiguous cases by a committee of experts) [4]. The beneficial outcome of survival without sensorimotor deficiency was considered as a secondary outcome. We assessed this in four populations: foetuses alive at maternal admission to hospital, live births, babies admitted to neonatal intensive care and survivors.

### Intensity of active perinatal care

**Fig. 1:**
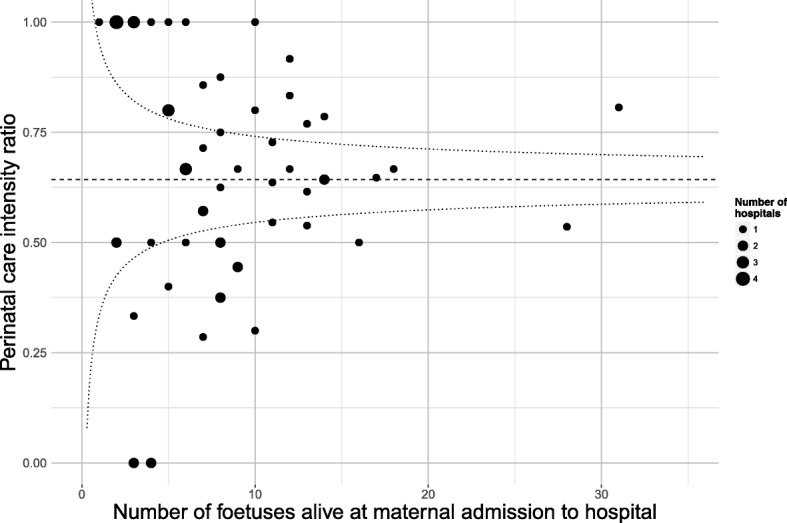
Intensity of perinatal care at 24–25 weeks gestation in French level 3 hospitals. Perinatal intensity is calculated as the ratio of babies born at 24–25 weeks gestational age who were admitted into neonatal intensive care divided by the number of foetuses delivered at the same gestational age who were alive at maternal admission to hospital or when the decision to perform caesarean section was made; weighted average intensity is indicated with a dashed line, 25*th* and 75*th* percentile limits with dotted lines

We categorised the care provided by teams at different hospitals into three groups using ‘perinatal intensity’ ratios based on the number of babies of 24–25 weeks gestation admitted into neonatal intensive care divided by the number of foetuses alive at maternal admission to hospital and subsequently delivered at 24–25 weeks gestation. We identified the 25*th* and 75*th* percentile limits around the average intensity, weighted according to the number of viable foetuses admitted to hospital [21]. This accounted for increased variability around the estimates for hospitals with few admissions (thus addressing the concern that the intensity ratio for the smaller hospitals may be imprecise). These limits were used to create ‘low’-, ‘medium’- and ‘high’-intensity groups of hospitals, as shown in Fig. 1; subjects were assigned according to their hospital of birth. Detailed methods are provided in Additional file 1.

### Potential explanatory variables

Data were also available for maternal, pregnancy and neonatal factors.

Maternal characteristics considered were as follows: age (less than 20, 20–24, 25–29, 30 and over), parity (number of previous viable births), country of birth (France or another country) and socioeconomic status (defined according to the highest occupational status of both parents or mother only if it was a single-parent family). In relation to the current pregnancy, there was information on fertility treatment, singleton or multiple pregnancy, foetal sex, presence of clinically diagnosed chorioamnionitis, whether there was preterm prolonged rupture of membranes (pPROM, defined as occurring more than 12 h prior to delivery), if there was a spontaneous onset of labour, length of maternal admission prior to delivery (days), gestational age at delivery (in completed weeks gestation) and foetal presentation. For babies, birth weight *z*-score (using French ‘EPOPé’ intrauterine growth curves [22]) was available.

Data relating to perinatal management (antenatal steroids, tocolysis, *in utero* transfer, magnesium sulphate, maternal antibiotic therapy, mode of delivery and neonatal resuscitation) were also available but were not used as they were considered to be constituents of the exposure.

### Statistical methods

Crude associations of the potential explanatory variables were identified through cross-tabulation with perinatal intensity levels. In order to assess the validity of our perinatal intensity level indicator, we also examined associations with the variables relating to perinatal management. Multilevel logistic regression analysis using clustering at the level of the hospital was then performed between the assigned intensity level and the outcome to provide an unadjusted estimate of the association. This model was amended by sequentially adding gestational age at delivery (model 2), multiple pregnancy status (model 3) and then extra variables considered to be potential confounders. These were identified a priori as potentially of importance: maternal age, family socio-economic status, fertility treatment during the current pregnancy, chorioamnionitis, pPROM, spontaneous labour, foetal sex and foetal size at delivery.

Analyses for sensorimotor deficiency were conducted for babies surviving to 2 years of age. We analysed both complete cases and, due to missing data, imputed data sets. For survival without sensorimotor deficiency, all analyses used imputed data. First, we used the entire population of foetuses alive at maternal admission. As longer term survival in this population is likely to be closely linked to the exposure, we repeated analyses in the populations of babies who were born alive and in those subsequently admitted into neonatal intensive care, as well as in those who survived to 2 years of age. Sensitivity analyses were performed for primary and secondary outcomes using a restricted population of only those babies that were delivered between 24 and 26 weeks gestational age. All investigations were conducted using R version 3.3.3 [23]. A *p* value < 0.05 was considered statistically significant throughout. Multiple imputation was performed using chained equations with the R package ‘mice’ [24] and included variables potentially predicting non-response or the outcome as described previously [4]; full details are in Additional file 2.

## Results

**Fig. 2:**
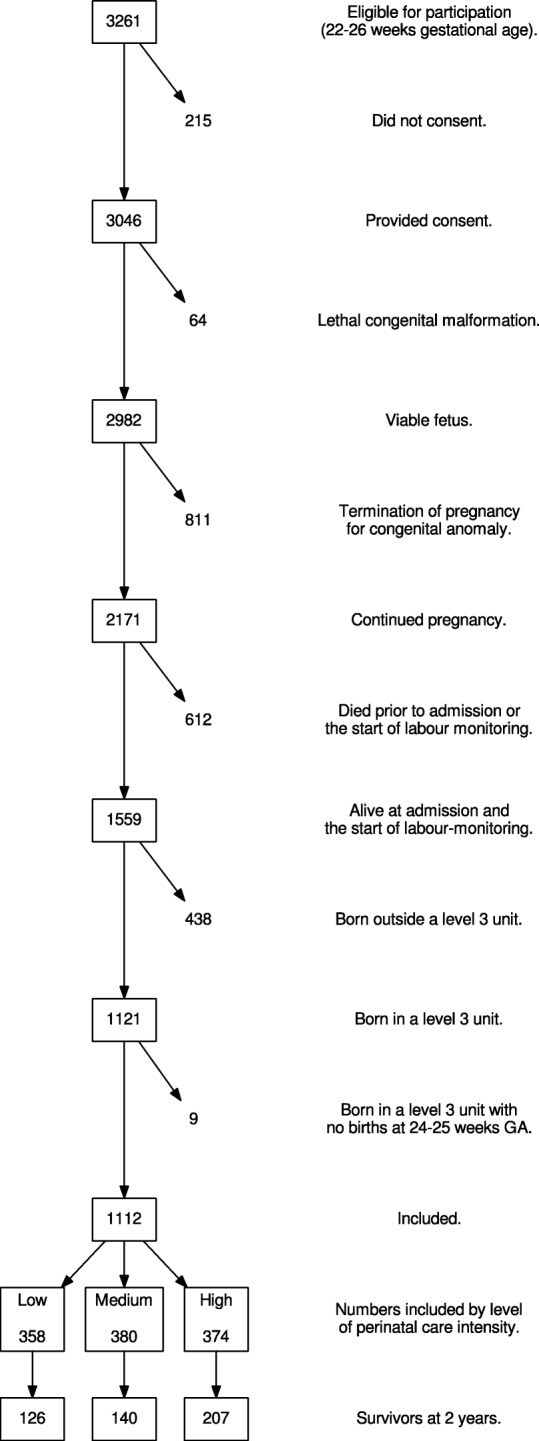
Study population. Flow chart of included study population: EPIPAGE-2 cohort at 2 years corrected age

Consent was provided for 3046 of 3261 births at 22–26 weeks gestation born in France. A further 1925 foetuses were excluded as they did not meet the inclusion criteria (Fig. 2), leaving 1121 who were alive at maternal admission to hospital and subsequently delivered at 22–26 weeks gestation. Of these, nine babies were born in one of three level 3 hospitals with no births at 24–25 weeks gestation. Thus, 1112 babies were included: 358 were born in one of the 19 hospitals categorised as low intensity, 380 in one of the 20 medium-intensity hospitals and 374 in the 23 high-intensity hospitals. Below 24 weeks gestation, there was only one survivor (born at 23 weeks and 6 days); hence, this child was included with those born at 24 weeks gestation for the main analyses but excluded from sensitivity analyses. The mean weighted intensity ratio was 64.3%, with hospital ratios ranging from 0 to 100% based on a range of 1 to 31 foetuses who were alive at maternal admission and subsequently delivered at 24 or 25 completed weeks gestation (Fig. 1).

There were important associations between perinatal intensity and perinatal management (Table 1). Antenatal steroids and tocolysis were more frequently administered in hospitals with a higher intensity level of perinatal care. Babies were more likely to be resuscitated in these hospitals. There were no differences in the administration of magnesium sulphate or proportions of women transferred *in utero*.

**Table 1:** Factors associated with active perinatal care according to perinatal care intensity level

Variable	Low		Medium		High		*p* value
	*n*	%	*n*	%	*n*	%	
Antenatal steroids (*N* = 1042)							
No	166	50.3	146	40.4	92	26.2	< 0.001
Yes	164	49.7	215	59.6	259	73.8	
In utero transfer (*N* = 1059)							
No	191	55.2	200	55.0	169	48.4	0.12
Yes	155	44.8	164	45.0	180	51.6	
Tocolysis (*N* = 1057)							
No	191	55.0	152	42.1	122	35.0	< 0.001
Yes	156	44.0	209	57.9	227	65.0	
MgSO4 usage (*N* = 1045)							
No	322	93.1	340	95.5	324	94.5	0.37
Yes	24	6.9	16	4.5	19	5.5	
Mode of delivery (*N* = 946)							
Caesarean	91	31.7	124	37.9	125	37.6	0.20
Vaginal	196	68.3	203	62.1	207	62.4	
Baby resuscitated (*N* = 803)							
No	74	31.2	37	14.1	32	10.6	< 0.001
Yes	163	68.8	226	85.9	271	89.4	

In univariate analysis, intensity of perinatal activity was strongly associated with gestational age at delivery, with hospitals of lower intensity having relatively higher numbers of babies born at 22 to 24 weeks gestation compared to hospitals with higher intensity. There was little difference in maternal length of stay prior to delivery, the numbers of singleton or multiple pregnancies or in maternal age profiles, but mothers were less likely to have had fertility treatment in low-intensity hospitals (*p* = 0.03): 14.7% compared to 20.5% and 22.1% in medium- and high-intensity units, respectively. Family socioeconomic status and the presence of pPROM also varied between groups. Complete data are shown in Table 2.

**Table 2:** Maternal and neonatal characteristics by perinatal care intensity level

Variable	Levels	Low		Medium		High		*p* value
		*n*	%	*n*	%	*n*	%	
Gestational age at delivery (*N* = 1112)								
	22	39	10.9	34	8.9	30	8.0	< 0.001
	23	43	12.0	50	13.2	36	9.6	
	24	88	24.6	79	20.8	50	13.4	
	25	75	21.0	103	27.1	115	30.8	
	26	113	31.6	114	30.0	143	38.2	
Maternal length of stay prior to delivery (*N* = 1059)								
	< 24 h	259	74.6	270	74.4	246	70.5	0.59
	24 to < 48 h	55	15.8	52	14.3	56	16.0	
	48 to < 72 h	16	4.6	20	5.5	18	5.2	
	72+ h	17	4.9	21	5.8	29	8.3	
Multiple pregnancy (*N* = 1112)								
	No	266	74.3	258	67.9	264	70.6	0.16
	Yes	92	25.7	122	32.1	110	29.4	
Foetal sex (*N* = 1105)								
	Male	178	50.3	180	47.6	178	47.7	0.72
	Female	176	49.7	198	52.4	195	52.3	
Maternal age group (*N* = 1105)								
	< 25 years	81	22.6	65	17.1	71	19.4	0.16
	25-29 years	99	27.6	121	31.8	119	32.4	
	30-34 years	94	26.3	123	32.4	105	28.6	
	≥ 35 years	84	23.5	71	18.7	72	19.6	
Mother born outside of France (*N* = 1033)								
	No	239	72.9	259	74.9	261	72.7	0.78
	Yes	89	27.1	87	25.1	98	27.3	
Single mother (*N* = 1044)								
	Yes	38	11.2	38	10.7	38	10.8	0.97
	No	300	88.8	317	89.3	313	89.2	
Family SES (*N* = 983)								
	Professional	64	20.0	49	14.9	95	28.4	0.01
	Intermediate	62	19.4	56	17.1	48	14.3	
	Administrative, public service, self-employed, students	75	23.4	101	30.8	77	23.0	
	Shop assistants, service workers	45	14.1	49	14.9	43	12.8	
	Manual workers	49	15.3	47	14.3	51	15.2	
	Unemployed	25	7.8	26	7.9	21	6.3	
Primiparous (*N* = 1101)								
	No	161	45.6	162	43.1	159	42.7	0.70
	Yes	192	54.4	214	56.9	213	57.3	
Maternal fertility treatment (*N* = 1069)								
	No	291	85.3	294	79.5	279	77.9	0.03
	Yes	50	14.7	76	20.5	79	22.1	
pPROM (*N* = 1053)								
	No	208	60.6	212	58.4	219	63.1	0.44
	Yes	135	39.4	151	41.6	128	36.9	
Chorioamnionitis diagnosed < 48 h to delivery (*N* = 930)								
	No	190	65.1	221	70.4	240	74.1	0.05
	Yes	102	34.9	93	29.6	84	25.9	
SGA (*N* = 1037)								
	Yes	83	25.0	77	21.8	88	25.1	0.50
	No	249	75.0	277	78.2	263	74.9	
Spontaneous labour (*N* = 908)								
	None	64	23.1	71	22.8	76	23.8	0.96
	Spontaneous	213	76.9	240	77.2	244	76.2	
Spontaneous rupture of membranes (*N* = 981)								
	No	130	39.8	130	40.0	138	42.0	0.82
	Yes	197	60.2	195	60.0	191	58.0	
Foetal presentation (*N* = 924)								
	Cephalic	139	50.0	177	55.8	183	55.6	0.19
	Breech	128	46.0	120	37.8	133	40.4	
	Other	11	4.0	20	6.3	13	4.0	

### Sensorimotor outcome at 2 years of age

Physician response rates to sensorimotor assessment at 2 years of age were 85.7%, 82.1% and 82.6% in the low-, medium- and high-intensity groups respectively. There were no statistically significant differences between the groups in the level of sensorimotor disability, with rates ranging from 5.8 to 7.0% (*p* = 0.9, Table 3). Complete case analysis did not demonstrate any differences in disability status for survivors who were born in hospitals of different intensity levels. Complete data were available for 394 cases for the baseline model and those adjusted for gestational age and multiple status. After including all a priori postulated factors, adjusted odds ratios (OR) among the 310 complete cases were 0.67 (95% confidence interval (CI) 0.17 to 2.69) for medium- and 0.81 (95% CI 0.23 to 2.81) for high-intensity hospitals (Table 4).

**Table 3:** Numbers and percentages of participants with confidence intervals by level of intensity

	Perinatal intensity level								
	Low			Medium			High		
	*n*	%	(95%CI)	*n*	%	(95%CI)	*n*	%	(95%CI)
Foetal admissions	358	–	–	380	–	–	374	–	–
Live births	243	67.9	(62.7–72.6)	274	72.1	(67.3–76.5)	308	82.4	(78.0–86.0)
Admitted to NICU	171	47.8	(42.5–53.1)	225	59.2	(54.1–64.2)	276	73.8	(69.0–78.1)
Alive at 2 years	126	35.2	(30.3–40.4)	140	36.8	(32.0–41.9)	207	55.3	(50.1–60.4)
CP (*n* responding)	108	–	–	115	–	–	171	–	–
CP/sensory deficiency	7	6.5	(2.9–13.4)	8	7.0	(3.3–13.7)	10	5.8	(3.0–10.8)
Imputed population*	126	–	–	140	–	–	207	–	–
CP/sensory deficiency*	–	6.9	(4.7–9.2)	–	7.8	(5.5–10.0)	–	7.3	(5.5–9.1)

*Imputed percentages were averaged across the 60 imputed data sets using Rubin’s rule [25]

**Table 4:** Sensorimotor outcomes at 2 years of age among survivors of babies born at 24–26 weeks gestation in medium- and high-intensity units compared to low-intensity units in France in 2011

Model	Number	Medium intensity		High intensity	
		OR	(95% CI)	OR	(95% CI)
Complete cases					
Unadjusted	394	1.08	(0.38–3.08)	0.90	(0.33–2.43)
Baseline + GA	394	0.84	(0.29–2.48)	0.72	(0.26–2.00)
Baseline + GA + multiple status	394	0.87	(0.29–2.62)	0.73	(0.26–2.07)
Baseline + extra variables	310	0.67	(0.17–2.69)	0.81	(0.23–2.81)
After multiple imputation					
Baseline	473	1.13	(0.41–3.17)	1.05	(0.39–2.79)
Baseline + GA	473	0.95	(0.33–2.73)	0.88	(0.32–2.40)
Baseline + GA + multiple status	473	1.02	(0.35–3.01)	0.92	(0.33–2.57)
Baseline + extra variables	473	0.93	(0.28–3.04)	1.04	(0.34–3.14)

*95% CI* 95% confidence interval, *GA* gestational age, *extra variables* GA + multiple status + foetal sex + maternal age + family SES + fertility treatment + chorioamnionitis + labour type + SGA + premature rupture of membranes

Following multiple imputation, the rates of sensorimotor deficiency were slightly higher than in the complete cases: 6.9% (95% CI 4.7 to 9.2), 7.8% (95% CI 5.5 to 10.0) and 7.3% (95% CI 5.5 to 9.1) corresponding to low-, medium- and high-intensity groups, respectively (Table 3). The baseline unadjusted OR was 1.13 (95% CI 0.41 to 3.17) in medium-intensity hospitals and 1.05 (95% CI 0.39 to 2.79 in high-intensity hospitals, both compared to hospitals with a low perinatal intensity level). There was little change after adjustment: the fully adjusted ORs were 0.93 (95% CI 0.28 to 3.04) and 1.04 (95% CI 0.34 to 3.14) in medium- and high-intensity centres, respectively. Full results are in Table 4.

### Morbidity-free survival

Compared to births in a low-intensity hospital, survival to 2 years of age without sensorimotor deficiency was higher in hospitals of high perinatal intensity in the population of foetuses who were alive at maternal admission to hospital. After multiple imputation, the unadjusted OR was 2.48 (95% CI 1.62 to 3.78). Results were attenuated by adjustment for gestational age to an OR of 2.15 (95% CI 1.37 to 3.37) and, following inclusion of all a priori considered potential confounders, remained similar. There was no difference for babies born in hospitals with a medium level of intensity compared to those born in low-intensity hospitals in this population.

Among live births, there was also improved morbidity-free survival in babies born in high-intensity hospitals: the unadjusted odds ratio (1.97, 95% CI 1.17 to 3.33) remaining significant after adjustment for all factors (adjusted OR 1.74, 95% CI 1.05 to 2.88). There was no evidence of a difference for babies born in a hospital of medium intensity.

When considering only those babies who were admitted into neonatal intensive care, there was no evidence of a difference in outcome for babies born in high- (adjusted OR 1.17, 95% CI 0.68 to 2.01) or medium- (adjusted OR 0.70, 95% CI 0.40 to 1.21) intensity hospitals compared to those born in a hospital of low-intensity perinatal care. Complete results for all populations are shown in Table 5.

**Table 5:** Survival without sensorimotor disability at 2 years of age in populations delivered at 22–26 weeks gestation in France in 2011

Population	Model	Medium intensity		High intensity	
		OR	(95% CI)	OR	(95% CI)
Foetuses	Baseline (unadjusted)	1.08	(0.71–1.64)	2.48	(1.62–3.78)
	Baseline + GA	1.06	(0.67–1.66)	2.15	(1.37–3.37)
	Baseline + GA + multiple status	1.00	(0.65–1.56)	2.12	(1.36–3.29)
	Baseline + GA + multiple status + extra factors	1.01	(0.63–1.61)	2.18	(1.37–3.46)
Live births	Baseline (unadjusted)	1.03	(0.60–1.74)	1.97	(1.17–3.33)
	Baseline + GA	0.94	(0.57–1.54)	1.77	(1.09–2.89)
	Baseline + GA + multiple status	0.89	(0.55–1.45)	1.75	(1.08–2.83)
	Baseline + GA + multiple status + extra factors	0.91	(0.54–1.51)	1.74	(1.05–2.88)
NICU admissions	Baseline (unadjusted)	0.59	(0.35–1.02)	1.02	(0.60–1.74)
	Baseline + GA	0.68	(0.40–1.17)	1.16	(0.68–1.97)
	Baseline + GA + multiple status	0.66	(0.39–1.13)	1.16	(0.69–1.96)
	Baseline + GA + multiple status + extra factors	0.70	(0.40–1.21)	1.17	(0.68–2.01)
Survivors	Baseline (unadjusted)	0.88	(0.32–2.47)	0.95	(0.36–2.54)
	Baseline + GA	1.06	(0.37–3.05)	1.14	(0.42–3.13)
	Baseline + GA + multiple status	0.98	(0.33–2.88)	1.08	(0.39–3.01)
	Baseline + GA + multiple status + extra factors	1.04	(0.32–3.38)	0.96	(0.32–2.86)

*Foetuses* foetuses (babies) alive at the onset of labour/maternal admission to hospital, *GA* gestational age, *extra factors* foetal sex + maternal age + family SES + fertility treatment + chorioamnionitis + labour type + SGA + premature rupture of membranes

### Sensitivity analyses

Results from sensitivity analyses using populations restricted to births from 24 to 26 weeks GA did not show any important differences from the main analyses. Tables corresponding to Tables 1, 2, 3, 4, and 5 are presented in Additional file 3.

## Discussion

### Principal findings

This study examined the impact of the intensity of perinatal care on outcomes at 2 years of age for extremely preterm babies born in French level 3 hospitals in 2011. Among survivors, there was no evidence of a difference in the adverse primary outcome of sensorimotor disability. There were, however, important differences in the beneficial outcome of survival without sensorimotor morbidity for babies born in hospitals of high-intensity perinatal care compared with those born in hospitals of low intensity among the populations of foetuses alive at admission to hospital and in live births, but no differences when considering babies admitted to neonatal intensive care. Results remained consistent in sensitivity analyses restricted to 24 to 26 weeks gestation.

### Strengths and limitations of this study

Strengths of our study include the fact it is a large, prospectively collected national cohort with standardised definitions of outcomes following international recommendations. Data collection was comprehensive [2, 4] with an overall follow-up rate of 83.3% for the sensorimotor outcome for the children included in this study. This is better than or similar to other population cohorts at a similar age [26, 27]. We used multiple imputation [24] to help mitigate this issue, but ideally, follow-up would be higher as the impact of missing data is difficult to ascertain: it is not possible to know whether children lost to follow-up are more or less likely to be impaired [28].

The measure of perinatal care intensity we used makes no assumptions about appropriate or ‘best’ practices. Instead, we assumed ‘active’ management would use any and all appropriate available techniques to ensure the baby survives. In the scenario we examined, this translates to hospitals providing treatment such that babies are subsequently admitted into neonatal intensive care. Following maternal admission to hospital, foetal deaths prior to delivery (stillbirths) or in the delivery room were therefore interpreted as a sign of inactive management. We restricted our measure of activity to include only births at 24 to 25 weeks gestational age as these are the ages when there was the greatest variability in practice in France in 2011: babies born before 24 weeks gestation almost uniformly did not receive active care, whereas at 26 weeks gestation, the inverse was true [15, 17].

That there were very few admissions at 24 to 25 weeks gestation in some hospitals, meaning that the indicator was partly based on small numbers, could be a weakness. We addressed the possible imprecision of the intensity ratio for these hospitals by weighting the mean around which the groups were constructed. A counter point is that we included almost all births at 22–26 weeks gestation occurring in level 3 hospitals in France. Only three of 65 units did not admit any women who would subsequently deliver at 24–25 weeks gestation—meaning we were unable to calculate perinatal intensity ratios. However, between them, there were only nine deliveries at 22–26 weeks gestation in these three hospitals.

The overall size of the cohort may also be perceived as a problem. In fact, the numbers in EPIPAGE-2 do not differ greatly from other cohorts. The first EPICure in 1995 included 4004 births under 26 weeks gestation with 308 survivors to 2.5 years of age in Great Britain and Ireland over 10 months [29, 30]; the second EPICure in 2006 included 2326 births below 27 weeks gestation with 1031 survivors to 3 years of age in England during 1 year [26, 31]; EXPRESS reported 774 births below 27 weeks gestation with 491 survivors at 2 years of age in Sweden during a 4-year period [27, 32]; and EPIPAGE-2 reported 2205 births below 27 weeks gestation with 545 survivors at 2 years in France in 8 months of 2011 [2, 4]. Compared to England in 2006, there was a higher proportion of babies born in a level 3 unit in France in 2011 (79.0% [2] compared to 56.4% in the EPICure 2 study [33]). For comparison, there are observational studies with larger numbers of subjects, but these tend to have been conducted by research networks without defined geographical coverage over a number of years.

We also note that the distribution of gestational age at delivery appears to differ between the intensity groups. This may be due to the fact that women admitted to high-intensity hospitals with threatened preterm labour are more likely than those admitted to low-intensity hospitals to have treatments aimed at prolonging the pregnancy—and of delivering a liveborn baby in good condition such that the baby is subsequently admitted to neonatal intensive care. This hypothesis is supported by the associations seen with active management treatments (e.g. tocolysis and antenatal steroids) which were more frequently used in women who delivered in high-intensity hospitals. However, there was no difference in overall length of maternal admission to hospital prior to delivery. An alternative hypothesis is, therefore, that the skewed distribution is linked to the construction of the indicator (as it uses babies born at both 24 and 25 weeks gestation). This does not explain differences in the numbers of babies born at 22, 23 or 26 weeks, though, so there may be a different reason or combination of reasons for the differences seen. We accounted for this potential bias by including gestational age in all adjusted analyses. Similarly, while there were no differences in the distribution of multiple pregnancies between intensity groups, we felt it was important to account for this in our models as there was potential for this to influence the ratios used to classify hospitals.

Another criticism might be that the indicator used as the exposure is contained within one of the outcomes (survival without sensorimotor disability), and thus, a positive association is to be expected. While this may be partly true—an ‘active’ hospital is one that has improved ‘perinatal’ survival (defined here as survival prior to admission to neonatal intensive care)—our definition of intensity only included foetuses subsequently delivered at 24 to 25 weeks gestation whereas the morbidity-free survival outcome also included deliveries occurring at 22, 23 and 26 weeks gestation. Consequently, the results were not certain, particularly given the high degree of variability in the numbers of babies born in each hospital at each week of gestation. Furthermore, intensity was measured at the hospital level but assigned to individual subjects. This is the same method and the same population as previously used by investigators in the EXPRESS study [14]. In that study, they used rates of obstetric treatments such as administration of antenatal steroids, tocolysis and caesarean section—all of which are strongly linked to improved survival prior to neonatal unit admission [34]—as contributory factors towards their ‘regional activity score’. The EXPRESS investigators additionally performed analyses in the live-born population, as did we. However, by investigating the population who were admitted to neonatal intensive care, our results indicate that survival differences only occur prior to intensive care unit admission without subsequent impact on morbidity status. In turn, this suggests that it is not possible to identify in the delivery room those babies who will have a better—or worse—long-term outcome. Finally, we note that in our study, survival was but a secondary outcome: there is no obvious link between our measure of perinatal intensity and the primary outcome of sensorimotor disability at 2 years of age.

### Study findings in context

Previous studies have investigated the relationship between the volume of admissions and outcome [33] or between measures of activity based on individual treatments such as administration of antenatal steroids, tocolysis, magnesium sulphate, caesarean section or postnatal attempts at resuscitation (for example, intubation or the administration of surfactant) [9, 14, 35, 36]. Such indicators are dependent upon the presenting circumstances of each patient and on local management protocols. They therefore may not be good indicators of differences in the intensity of active perinatal management between hospitals or regions due to differences in population make-up or varying protocols. For example, tocolysis may be of use in women who have pPROM but is not indicated in the absence of active labour. Antenatal steroids are recommended for threatened preterm delivery [37], but there may be insufficient time prior to delivery for administration. Both of these indicators consequently vary according to the presenting population. There are also differences postnatally. Some hospitals routinely intubate babies born at extremely preterm gestations. Others seek to avoid intubation altogether during the first 72 h of life, preferring to utilise techniques such as continuous positive airway pressure along with less invasive surfactant administration via a narrow-bore endotracheal catheter during spontaneous respiration [38]. The validity of our indicator was additionally highlighted by showing strong relationships with such perinatal management factors.

Our investigation focused on the intensity of active perinatal care in hospitals, thus trying to ascertain the effect from groups of professionals who work together on a daily basis. Importantly, this includes both obstetric and neonatal elements, rather than just obstetric *or* neonatal. We are thus better able to answer the question, ‘does intensity of active perinatal care improve longer term prognosis?’ The simplicity of the indicator means it is easily transferable to other situations as vital status measures are commonly made. For example, it can be applied using different baseline gestational ages according to local attitudes towards extremely preterm delivery. It could be applied to regions or networks instead of hospitals and is also easily transportable between cohorts. It could also be applicable in other situations, such as the admission of patients with head injury via the emergency room to intensive care or adults suffering in-hospital cardiac arrest who are subsequently admitted to a coronary care unit.

Although there was only one survivor born below 24 weeks GA, attitudes in France towards extreme preterm birth are not dissimilar to some other European countries [7]. Recent studies from the Netherlands demonstrate most variation in attitudes among obstetricians and neonatologists occurring at 24 and 25 weeks gestation [39], with significant mortality at these gestations [40]. A study comparing five different European regions showed the largest differences in survival for babies born at 500 g or more at 24 weeks gestation; results for births at 23 and 25 weeks and below 500 g were much more similar [41]. More importantly, perhaps, there have been changes over time in attitudes to extremely preterm birth across Europe, and hospitals that have actively changed their policies seem to have experienced larger survival gains [42]. We also note that our findings are consistent with those from studies in Sweden [14] and the USA [9].

### Conclusion

This study described a novel measure of the intensity of perinatal care comprised of common vital measures which could be applied broadly elsewhere. In this population of women delivering at extremely preterm gestations, we found no evidence of increased sensorimotor impairment at 2 years of age for babies born in hospitals with a higher intensity of active perinatal management. We demonstrated an important improvement in survival without sensorimotor disability in populations of foetuses alive at maternal admission to hospital and in live births. Together, these results indicate that hospitals with higher perinatal intensity levels improve survival without increasing sensorimotor morbidity at 2 years of age. They support findings from other populations that outcomes are better following higher intensity treatment at birth and strengthen the case that more aggressive treatment does not lead to increased levels of morbidity.

## Additional files

Additional file 1 S1 Appendix. Creating a ratio to measure intensity of active perinatal care. (PDF 143 kb)

Additional file 2 S2 Appendix. Methods for multiple imputation. (PDF 84 kb)

Additional file 3 S3 Appendix. Results from sensitivity analyses using populations delivered at 24–26 weeks gestation. (PDF 108 kb)

Additional file 4 S4 Appendix. STROBE checklist. (PDF 142 kb)

## Abbreviations

GA: Gestational age
