# Supplementary material for: Intensity of perinatal care, extreme prematurity and sensorimotor outcome at 2 years corrected age: evidence from the EPIPAGE-2 cohort study
Source: BMC Med. 2018 Dec 5;16:227. doi: 10.1186/s12916-018-1206-4 (PMC6280378; doi:10.1186/s12916-018-1206-4)
Supplement: Supplementary file 1 — S1 Appendix. Creating a ratio to measure intensity of active perinatal care. (PDF 143 kb) [file 12916_2018_1206_MOESM1_ESM.pdf]

RESEARCH

S1 Appendix: construction of the 'intensity of active perinatal care' ratio

Andrei S Morgan[1,2,3*], Laurence Foix L'Helias[1,4,5], Caroline Diguisto[1,6,7], Laetitia Marchand-Martin[1], Monique Kaminski[1], Babak Khoshnood[1], Jennifer Zeitlin[1], Gérard Bréart[1], Xavier Durrmeyer[1,8], François Goffinet[1,9] and Pierre-Yves Ancel[1,10]

*Correspondence: andrei.morgan@inserm.fr
[1]INSERM UMR 1153, Obstetrical, Perinatal and Pediatric Epidemiology Research Team (EPOPé), Centre for Epidemiology and Statistics Sorbonne Paris Cité, DHU Risks in Pregnancy, Paris Descartes University, Hôpital Tenon, Rue de la Chine, 75020 Paris, France
Full list of author information is available at the end of the article

We categorised hospitals into three levels, low, medium and high, according to a weighted calculation[1]of "perinatal intensity" based on admissions at 24 to 25 weeks' gestation.

First, each individual hospital was assigned an activity ratio according to the number of babies admitted into a neonatal intensive care unit at 24 to 25 weeks' gestation divided by the number of fetuses alive at maternal admission to hospital at the same gestations (equation 1).

$$\text{Activity ratio } (p_i) = \frac{\text{Number of babies admitted into NICU}}{\text{Number of foetuses alive at maternal admission to hospital}} \quad (1)$$

Using these ratios, the mean activity level across all included hospitals was obtained using formula shown in equation 2, where P_w is the overall weighted mean, p_i is the activity ratio in hospital i, and w_i is the weighting factor for hospital i:

$$P_w = \frac{\sum p_i w_i}{\sum w_i} \quad (2)$$

The weighting factors for individual hospitals were obtained using the formula shown in equation 3.

$$w_i = \frac{1}{\hat{\sigma}_p^2 + \frac{(\bar{p}(1-\bar{p})-\hat{\sigma}_p^2)}{n_i}} \quad (3)$$

In this equation, $\bar{p}$ represents the unweighted mean activity ratio of all hospitals (obtained simply by summation of all the ratios and dividing by the total number of hospitals), and $\hat{\sigma}_p^2$ is the estimated standard deviation, which is obtained from the following equation:

$$\hat{\sigma}_p^2 = \frac{\sum(p_i - \bar{p})^2}{k-1} - \frac{\sum \frac{p_i(1-p_i)}{n_i}}{k} \quad (4)$$

Here, again, $\bar{p}$ is the unweighted mean activity ratio, p_i is the activity ratio for hospital i, n_i is the number of fetuses alive at maternal admission to hospital in hospital i, and k is the total number of hospitals.

Having calculated the mean activity level, 25th and 75th percentiles were obtained for different numbers of fetuses alive at maternal admission to hospital using equation 5:

$$\text{25th/75th percentiles} = P_w \pm 0.675\left(\frac{\sqrt{\hat{\sigma}_p^2}}{\sqrt{n}}\right) \quad (5)$$

where n is the number of foetuses admitted into hospital and $\hat{\sigma}_p^2$ is defined by equation 4. This enabled individual hospitals to be compared to the percentiles, and consequently permitting allocation to one of the three potential groups created (see figure 1 in the main article).

Author details

[1]INSERM UMR 1153, Obstetrical, Perinatal and Pediatric Epidemiology Research Team (EPOPé), Centre for Epidemiology and Statistics Sorbonne Paris Cité, DHU Risks in Pregnancy, Paris Descartes University, Hôpital Tenon, Rue de la Chine, 75020 Paris, France. [2]Institute for Womens' Health, UCL, 74 Huntley Street, WC1E 6AU, London, UK. [3]SAMU 93 - SMUR Pédiatrique, CHI André Gregoire, Groupe Hospitalier Universitaire Paris Seine-Saint-Denis, Assistance Publique des Hôpitaux de Paris, Montreuil, France. [4]UPMC Université Paris 6, Sorbonne Universités, Paris, France. [5] Service de Néonatologie, Hopital Armand Trousseau, Assistance Publique des Hôpitaux de Paris, Paris, France. [6] Maternité Olympe de Gouges, Centre Hospitalier Regional Universitaire Tours, Tours, France. [7] Université François Rabelais, Tours, France. [8] Service de Médecine Néonatale, Centre Hospitalier Intercommunal de Creteil, Clinical Research Center CHI Créteil, Créteil, France. [9] Maternité Port-Royal, University Paris-Descartes, Hôpitaux Universitaires Paris Centre, Assistance Publique des Hôpitaux de Paris, Paris, France. [10] URC CIC P1419, DHU Risk in Pregnancy, Cochin Hotel Dieu, Assistance Publique des Hôpitaux de Paris, Paris, France.

References

1. Laird, N.M., Mosteller, F.: Some statistical methods for combining experimental results. International Journal of Technology Assessment in Health Care **6**(1), 5–30 (1990)
