# Supplementary material for: Intensity of perinatal care, extreme prematurity and sensorimotor outcome at 2 years corrected age: evidence from the EPIPAGE-2 cohort study
Source: BMC Med. 2018 Dec 5;16:227. doi: 10.1186/s12916-018-1206-4 (PMC6280378; doi:10.1186/s12916-018-1206-4)
Supplement: Supplementary file 2 — S2 Appendix. Methods for multiple imputation. (PDF 84 kb) [file 12916_2018_1206_MOESM2_ESM.pdf]

RESEARCH

S2 Appendix: Multiple imputation methods

Andrei S Morgan[1,2,3*], Laurence Foix L'Helias[1,4,5], Caroline Diguisto[1,6,7], Laetitia Marchand-Martin[1], Monique Kaminski[1], Babak Khoshnood[1], Jennifer Zeitlin[1], Gérard Bréart[1], Xavier Durrmeyer[1,8], François Goffinet[1,9] and Pierre-Yves Ancel[1,10]

*Correspondence:
andrei.morgan@inserm.fr
[1]INSERM UMR 1153, Obstetrical, Perinatal and Pediatric Epidemiology Research Team (EPOPé), Centre for Epidemiology and Statistics Sorbonne Paris Cité, DHU Risks in Pregnancy, Paris Descartes University, Hôpital Tenon, Rue de la Chine, 75020 Paris, France
Full list of author information is available at the end of the article

We used multiple imputation due to incomplete responses in physician questionnaires at two years of age. This was performed using chained equations with the R package 'mice',[1] imputing data for outcomes and other covariates. Imputation model variables included the exposure, two year outcomes (sensorimotor disability and survival status), maternal variables (age, parity, country of birth, socioeconomic status), pregnancy variables (fertility treatment, multiple status, fetal sex, chorioamnionitis, prolonged rupture of membranes, spontaneous labour, gestational age at delivery), birth weight z-score, neonatal morbidities (bronchopulmonary dysplasia, necrotising enterocolitis and retinopathy of prematurity) as well as usage of postnatal steroids, whether the baby received maternal breast milk at discharge, and whether there was a severe congenital brain malformation. We also included data relating to the five domains (communication, gross motor, fine motor, problem-solving and personal-social) of the Ages and Stages Questionnaire (ASQ)[2] if completed between 22 and 26 months corrected age in children without cerebral palsy or neurosensory impairment (deafness and blindness), and who did not have a severe brain malformation. Data for sensorimotor deficiency and ASQ status were only imputed for children who survived to two years of age. We generated 60 independent data sets using 30 iterations each; data were pooled according to Rubin's rule.[3]

Author details
[1]INSERM UMR 1153, Obstetrical, Perinatal and Pediatric Epidemiology Research Team (EPOPé), Centre for Epidemiology and Statistics Sorbonne Paris Cité, DHU Risks in Pregnancy, Paris Descartes University, Hôpital Tenon, Rue de la Chine, 75020 Paris, France. [2]Institute for Womens' Health, UCL, 74 Huntley Street, WC1E 6AU, London, UK. [3]SAMU 93 - SMUR Pédiatrique, CHI André Gregoire, Groupe Hospitalier Universitaire Paris Seine-Saint-Denis, Assistance Publique des Hôpitaux de Paris, Montreuil, France. [4]UPMC Université Paris 6, Sorbonne Universités, Paris, France. [5] Service de Néonatologie, Hopital Armand Trousseau, Assistance Publique des Hôpitaux de Paris, Paris, France. [6] Maternité Olympe de Gouges, Centre Hospitalier Regional Universitaire Tours, Tours, France. [7] Université François Rabelais, Tours, France. [8] Service de Médecine Néonatale, Centre Hospitalier Intercommunal de Creteil, Clinical Research Center CHI Créteil, Créteil, France. [9] Maternité Port-Royal, University Paris-Descartes, Hôpitaux Universitaires Paris Centre, Assistance Publique des Hôpitaux de Paris, Paris, France. [10] URC CIC P1419, DHU Risk in Pregnancy, Cochin Hotel Dieu, Assistance Publique des Hôpitaux de Paris, Paris, France.

References

1. van Buuren, S., Groothuis-Oudshoorn, K.: mice: Multivariate Imputation by Chained Equations in R. Journal of Statistical Software, Articles **45**(3), 1–67 (2011). doi:10.18637/jss.v045.i03
2. Squires, J., Bricker, D., Twombly, E., Nickel, R., Clifford, J., Murphy, K., Hoselton, R., Potter, L., Mounts, L., Farrell, J.: Ages & Stages Questionnaires, Third Edition (ASQ-3). Brookes Publishing, ??? (2009)
3. Rubin, D.B.: Multiple Imputation for Nonresponse in Surveys. John Wiley & Sons, Inc., USA (1987). doi:10.1002/9780470316696.fmatter

Table 1: Multiple imputation variables: methods and missingness

Variable	Variable type	Model for data prediction	Percentage of values missing			
			foetuses	live births	NICU admissions	survivors
Hospital of birth	Categorical	No missing data	0%	0%	0%	0%
Intensity of perinatal care group	Categorical (3 categories)	No missing data	0%	0%	0%	0%
Survival status	Categorical (6 categories)	No missing data	0%	0%	0%	0%
Gestational age (weeks)	Numeric	No missing data	0%	0%	0%	0%
Multiple pregnancy status	Binary	No missing data	0%	0%	0%	0%
Fetal sex	Binary	Logistic regression	0.6%	0.1%	0%	0%
Maternal age	Categorical (4 categories)	Ordered logit model	0.6%	0.2%	0%	0%
Parents' socioeconomic status	Categorical (6 categories)	Multinomial logit model	11.5%	9.6%	8.8%	6.6%
Maternal fertility treatment	Binary	Logistic regression	3.8%	3.6%	3.4%	3.4%
Chorioamnionitis	Binary	Logistic regression	16.2%	11.8%	11.0%	10.1%
Spontaneous labour	Binary	Logistic regression	18.2%	5.1%	4.2%	4.0%
Birth weight z-score	Numeric	Predictive mean matching	6.7%	0.4%	0%	0%
Premature rupture of membranes	Binary	Logistic regression	5.3%	1.2%	0.7%	0.4%
Maternal origin	Binary	Logistic regression	7.0%	5.2%	3.7%	1.3%
Primiparous	Binary	Logistic regression	1.0%	1.2%	1.2%	1.3%
Postnatal steroids	Binary	Logistic regression	41.8%	22.1%	4.3%	3.2%
Severe bronchopulmonary dypslasia	Binary	Logistic regression	51.3%	34.9%	20.1%	7.0%
Severe necrotising enterocolitis	Binary	Logistic regression	41.0%	21.0%	3.0%	0.6%
Severe retinopathy of prematurity	Binary	Logistic regression	40.4%	20.1%	1.9%	1.1%
Breastmilk at discharge	Binary	Logistic regression	59.9%	46.5%	34.4%	8.0%
Severe cerebral lesions	Binary	Logistic regression	40.9%	20.8%	2.8%	0.2%
Sensorimotor deficiency at 2 years CA	Binary	Logistic regression	64.0%	52.2%	41.4%	16.7%
ASQ Communication score below threshold at 2 years CA	Binary	Logistic regression	71.5%	62.3%	53.7%	34.2%
ASQ gross motor score below threshold at 2 years CA	Binary	Logistic regression	71.8%	62.8%	54.3%	35.1%
ASQ fine motor score below threshold at 2 years CA	Binary	Logistic regression	71.8%	62.8%	54.3%	35.1%
ASQ problem-solving score below threshold at 2 years CA	Binary	Logistic regression	72.3%	63.5%	55.2%	36.4%
ASQ personal-social score below threshold at 2 years CA	Binary	Logistic regression	72.0%	63.0%	54.6%	35.5%
