# Supplementary material for: Intensity of perinatal care, extreme prematurity and sensorimotor outcome at 2 years corrected age: evidence from the EPIPAGE-2 cohort study
Source: BMC Med. 2018 Dec 5;16:227. doi: 10.1186/s12916-018-1206-4 (PMC6280378; doi:10.1186/s12916-018-1206-4)
Supplement: Supplementary file 3 — S3 Appendix. Results from sensitivity analyses using populations delivered at 24–26 weeks gestation. (PDF 108 kb) [file 12916_2018_1206_MOESM3_ESM.pdf]

RESEARCH

S3 Appendix: sensitivity analyses using a baseline of 24 to 26 weeks gestational age

Andrei S Morgan[1,2,3*], Laurence Foix L'Helias[1,4,5], Caroline Diguisto[1,6,7], Laetitia Marchand-Martin[1], Monique Kaminski[1], Babak Khoshnood[1], Jennifer Zeitlin[1], Gérard Bréart[1], Xavier Durrmeyer[1,8], François Goffinet[1,9] and Pierre-Yves Ancel[1,10]

*Correspondence: andrei.morgan@inserm.fr
[1]INSERM UMR 1153, Obstetrical, Perinatal and Pediatric Epidemiology Research Team (EPOPé), Centre for Epidemiology and Statistics Sorbonne Paris Cité, DHU Risks in Pregnancy, Paris Descartes University, Hôpital Tenon, Rue de la Chine, 75020 Paris, France
Full list of author information is available at the end of the article

Table 1: Factors associated with active perinatal care according to perinatal care intensity level.

Variable		Low		Medium		High		P value
		n	%	n	%	n	%	
Antenatal steroids (N=838)								
	No	92	36.08	76	26.48	41	13.85	<0.001
	Yes	163	63.92	211	73.52	255	86.15	
In utero transfer (N=853)								
	No	129	48.31	136	46.9	126	42.57	0.356
	Yes	138	51.69	154	53.1	170	57.43	
Tocolysis (N=852)								
	No	137	51.12	103	35.76	85	28.72	<0.001
	Yes	131	48.88	185	64.24	211	71.28	
MgSO4 usage (N=839)								
	No	243	91.01	269	95.39	271	93.45	0.12
	Yes	24	8.99	13	4.61	19	6.55	
Mode of delivery (N=786)								
	Caesarean	91	39.22	121	45.32	121	42.16	0.388
	Vaginal	141	60.78	146	54.68	166	57.84	
Baby resuscitated (N=728)								
	No	46	22.01	16	6.72	15	5.34	<0.001
	Yes	163	77.99	222	93.28	266	94.66	

Table 2: Maternal and neonatal characteristics by perinatal care intensity level.

Variable	Levels	Low		Medium		High		P value
		n	%	n	%	n	%	
Gestational age (N=880)								
	24	88	31.88	79	26.69	50	16.23	<0.001
	25	75	27.17	103	34.8	115	37.34	
	26	113	40.94	114	38.51	143	46.43	
Multiple pregnancy (N=880)								
	no	201	72.83	198	66.89	216	70.13	0.301
	yes	75	27.17	98	33.11	92	29.87	
Fetal sex (N=878)								
	male	142	51.64	139	47.12	146	47.4	0.484
	female	133	48.36	156	52.88	162	52.6	
Maternal age group (N=878)								
	<25 years	62	22.46	48	16.22	56	18.3	0.201
	25-29 years	77	27.9	96	32.43	106	34.64	
	30-34 years	72	26.09	94	31.76	86	28.1	
	>=35 years	65	23.55	58	19.59	58	18.95	
Mother born outside of France (N=830)								
	No	182	71.65	212	77.37	220	72.85	0.279
	Yes	72	28.35	62	22.63	82	27.15	
Single mother (N=830)								
	Yes	30	11.41	31	11.19	28	9.66	0.764
	No	233	88.59	246	88.81	262	90.34	
Family SES (N=780)								
	Professional	57	23.27	42	16.28	76	27.44	0.156
	Intermediate	47	19.18	47	18.22	42	15.16	
	Administrative, public service, self-employed, students	59	24.08	79	30.62	66	23.83	
	Shop assistants, service workers	34	13.88	32	12.4	37	13.36	
	Manual workers	34	13.88	37	14.34	42	15.16	
	Unemployed	14	5.71	21	8.14	14	5.05	
Primiparous (N=870)								
	No	122	44.85	129	44.18	132	43.14	0.916
	Yes	150	55.15	163	55.82	174	56.86	
Maternal fertility treatment (N=841)								
	No	216	83.08	221	76.74	222	75.77	0.081
	Yes	44	16.92	67	23.26	71	24.23	
pPROM (N=849)								
	No	158	59.4	165	57.09	182	61.9	0.496
	Yes	108	40.6	124	42.91	112	38.1	
Chorioamnionitis diagnosed <48hrs to delivery (N=751)								
	No	147	65.33	172	68.53	207	75.27	0.044
	Yes	78	34.67	79	31.47	68	24.73	
SGA (N=846)								
	Yes	69	26.54	63	22.11	80	26.58	0.368
	No	191	73.46	222	77.89	221	73.42	
Spontaneous labour (N=758)								
	none	63	28.51	68	26.56	75	26.69	0.869
	spontaneous	158	71.49	188	73.44	206	73.31	
Spontaneous rupture of membranes (N=785)								
	No	104	41.77	104	40.47	115	41.22	0.956
	Yes	145	58.23	153	59.53	164	58.78	
Foetal presentation (N=770)								
	cephalic	119	53.36	144	55.17	155	54.2	0.203
	breech	96	43.05	97	37.16	119	41.61	
	other	8	3.59	20	7.66	12	4.2	

Table 3: Numbers and percentages of participants with confidence intervals by level of intensity.

	Perinatal intensity level								
	Low			Medium			High		
	n	%	(95%CI)	n	%	(95%CI)	n	%	(95%CI)
Fetal admissions	276	–	—	296	–	—	308	–	—
Live births	215	77.9	(72.4 – 82.6)	247	83.4	(78.6 – 87.4)	286	92.9	(89.2 – 95.4)
Admitted to NICU	171	62.0	(55.9 – 67.7)	221	74.7	(69.2 – 79.4)	273	88.6	(84.4 – 91.9)
Alive at 2 years	126	45.7	(39.7 – 51.7)	140	47.3	(41.5 – 53.2)	206	66.9	(61.3 – 72.1)
CP (n responding)	108	–	—	115	–	—	170	–	—
CP/sensory deficiency	7	6.5	(2.9 – 13.4)	8	7.0	(3.3 – 13.7)	10	5.9	(3.0 – 10.9)
**Imputed population**	126	–	—	140	–	—	207	–	—
CP/Sensory deficiency	–	6.9	(4.7 – 9.2)	–	7.7	(5.4 – 9.9)	–	7.2	(5.4 – 9.0)

Imputed percentages were averaged across the 60 imputed data sets using Rubin's rule.[1]

Table 4: Sensorimotor outcomes at two years of age amongst survivors of babies born at 24-26 weeks gestation in medium and high intensity units compared to low intensity units in France in 2011.

**Model**	**n**	**Medium intensity**		**High intensity**	
		**OR**	**(95% CI)**	**OR**	**(95% CI)**
**Complete cases**					
Unadjusted	393	1.08	(0.38 – 3.08)	0.90	(0.33 – 2.45)
Baseline + GA	393	0.84	(0.28 – 2.47)	0.73	(0.26 – 2.02)
Baseline + GA + multiple status	393	0.86	(0.28 – 2.61)	0.74	(0.26 – 2.11)
Baseline + extra variables	309	0.67	(0.17 – 2.68)	0.82	(0.24 – 2.82)
**After multiple imputation**					
Baseline	472	1.12	(0.40 – 3.15)	1.04	(0.39 – 2.72)
Baseline + GA	472	0.92	(0.32 – 2.68)	0.87	(0.32 – 2.34)
Baseline + GA + multiple status	472	1.00	(0.34 – 2.96)	0.92	(0.34 – 2.50)
Baseline + extra variables	472	0.91	(0.28 – 2.99)	1.02	(0.35 – 2.99)

95% CI: 95% confidence interval. GA: gestational age. Extra variables: GA + multiple status + fetal sex + maternal age + family SES + fertility treatment + chorioamnionitis + labour type + SGA + premature rupture of membranes.

Table 5: **Survival without sensorimotor disability at two years of age in populations delivered at 24-26 weeks gestation.**

Population	Model	Medium intensity		High intensity	
		OR	(95% CI)	OR	(95% CI)
Fetuses	Baseline (unadjusted)	1.08	(0.69 – 1.68)	2.40	(1.53 – 3.78)
	Baseline + GA	1.05	(0.68 – 1.62)	2.09	(1.35 – 3.23)
	Baseline + GA + multiple status	1.00	(0.65 – 1.53)	2.06	(1.34 – 3.15)
	Baseline + GA + multiple status + extra factors	1.01	(0.65 – 1.59)	2.14	(1.37 – 3.34)
Live births	Baseline (unadjusted)	0.94	(0.57 – 1.54)	1.77	(1.08 – 2.90)
	Baseline + GA	0.92	(0.57 – 1.48)	1.74	(1.08 – 2.81)
	Baseline + GA + multiple status	0.87	(0.55 – 1.39)	1.71	(1.07 – 2.73)
	Baseline + GA + multiple status + extra factors	0.90	(0.56 – 1.46)	1.74	(1.08 – 2.82)
NICU admissions	Baseline (unadjusted)	0.61	(0.36 – 1.05)	1.04	(0.61 – 1.76)
	Baseline + GA	0.69	(0.41 – 1.16)	1.16	(0.69 – 1.94)
	Baseline + GA + multiple status	0.66	(0.40 – 1.12)	1.16	(0.69 – 1.93)
	Baseline + GA + multiple status + extra factors	0.70	(0.41 – 1.21)	1.16	(0.68 – 1.98)
Survivors	Baseline (unadjusted)	0.90	(0.32 – 2.53)	0.97	(0.37 – 2.54)
	Baseline + GA	1.08	(0.37 – 3.14)	1.15	(0.43 – 3.10)
	Baseline + GA + multiple status	1.00	(0.34 – 2.98)	1.09	(0.40 – 2.97)
	Baseline + GA + multiple status + extra factors	1.06	(0.32 – 3.45)	0.97	(0.33 – 2.83)

Fetuses: fetuses (babies) alive at the onset of labour/maternal admission to hospital.
GA: gestational age. Extra factors: fetal sex + maternal age + family SES + fertility treatment + chorioamnionitis + labour type + SGA + premature rupture of membranes.

Author details
[1]INSERM UMR 1153, Obstetrical, Perinatal and Pediatric Epidemiology Research Team (EPOPé), Centre for Epidemiology and Statistics Sorbonne Paris Cité, DHU Risks in Pregnancy, Paris Descartes University, Hôpital Tenon, Rue de la Chine, 75020 Paris, France. [2]Institute for Womens' Health, UCL, 74 Huntley Street, WC1E 6AU, London, UK. [3]SAMU 93 - SMUR Pédiatrique, CHI André Gregoire, Groupe Hospitalier Universitaire Paris Seine-Saint-Denis, Assistance Publique des Hôpitaux de Paris, Montreuil, France. [4]UPMC Université Paris 6, Sorbonne Universités, Paris, France. [5] Service de Néonatologie, Hopital Armand Trousseau, Assistance Publique des Hôpitaux de Paris, Paris, France. [6] Maternité Olympe de Gouges, Centre Hospitalier Regional Universitaire Tours, Tours, France. [7] Université François Rabelais, Tours, France. [8] Service de Médecine Néonatale, Centre Hospitalier Intercommunal de Creteil, Clinical Research Center CHI Créteil, Créteil, France. [9] Maternité Port-Royal, University Paris-Descartes, Hôpitaux Universitaires Paris Centre, Assistance Publique des Hôpitaux de Paris, Paris, France. [10] URC CIC P1419, DHU Risk in Pregnancy, Cochin Hotel Dieu, Assistance Publique des Hôpitaux de Paris, Paris, France.

References

1. Rubin, D.B.: Multiple Imputation for Nonresponse in Surveys. John Wiley & Sons, Inc., USA (1987). doi:10.1002/9780470316696.fmatter
