# Supplementary material for: Intensity of perinatal care, extreme prematurity and sensorimotor outcome at 2 years corrected age: evidence from the EPIPAGE-2 cohort study
Source: BMC Med. 2018 Dec 5;16:227. doi: 10.1186/s12916-018-1206-4 (PMC6280378; doi:10.1186/s12916-018-1206-4)
Supplement: Supplementary file 4 — S4 Appendix. STROBE checklist. (PDF 142 kb) [file 12916_2018_1206_MOESM4_ESM.pdf]

RESEARCH

S4 Appendix: STROBE checklist

Andrei S Morgan[1,2,3*], Laurence Foix L'Helias[1,4,5], Caroline Diguisto[1,6,7], Laetitia Marchand-Martin[1], Monique Kaminski[1], Babak Khoshnood[1], Jennifer Zeitlin[1], Gérard Bréart[1], Xavier Durrmeyer[1,8], François Goffinet[1,9] and Pierre-Yves Ancel[1,10]

*Correspondence: andrei.morgan@inserm.fr
[1]INSERM UMR 1153, Obstetrical, Perinatal and Pediatric Epidemiology Research Team (EPOPé), Centre for Epidemiology and Statistics Sorbonne Paris Cité, DHU Risks in Pregnancy, Paris Descartes University, Hôpital Tenon, Rue de la Chine, 75020 Paris, France
Full list of author information is available at the end of the article

The following table contains the STROBE checklist of items to be included in reports of cohort studies[1] alongside a reference to where in the article the information may be found.

STROBE checklist for "Intensity of perinatal care and outcomes for extremely premature babies at two years corrected age: evidence from the EPIPAGE-2 cohort study"

	Item No	Recommendation	Section (notes)
**Title and abstract**	1	(a) Indicate the study's design with a commonly used term in the title or the abstract	Title ("cohort study")
		(b) Provide in the abstract an informative and balanced summary of what was done and what was found	Abstract: Methods and Results sections
**Introduction**			
Background/ rationale	2	Explain the scientific background and rationale for the investigation being reported	Background (paragraphs 1 to 3)
Objectives	3	State specific objectives, including any prespecified hypotheses	Background (final paragraph)
**Methods**			
Study design	4	Present key elements of study design early in the paper	Methods section
Setting	5	Describe the setting, locations, and relevant dates, including periods of recruitment, exposure, follow-up, and data collection	Methods ("Study population" section)

Continued on next page...

Strobe checklist (continued)

	Item No	Recommendation	Section (notes)
Participants	6	(a) Give the eligibility criteria, and the sources and methods of selection of participants. Describe methods of follow-up	Methods ("Study population" and "Outcomes" sections)
		(b) For matched studies, give matching criteria and number of exposed and unexposed	N/A
Variables	7	Clearly define all outcomes, exposures, predictors, potential confounders, and effect modifiers. Give diagnostic criteria, if applicable	Methods (sections on Outcomes, Intensity of perinatal care, Potential explanatory variables. Details are also provided on variables used for Multiple imputation S2 appendix; S1 appendix (contains detailed information on construction of the exposure variable)
Data sources/ measurement	8	For each variable of interest, give sources of data and details of methods of assessment (measurement). Describe comparability of assessment methods if there is more than one group.	Methods (sections on Outcomes, Intensity of perinatal care, Potential explanatory variables. Details are also provided on variables used for multiple imputation in S2 appendix)
Bias	9	Describe any efforts to address potential sources of bias	Methods ("Statistical methods" section and S2 appendix)
Study size	10	Explain how the study size was arrived at	Methods ("Study population") and Figure 2)
Quantitative variables	11	Explain how quantitative variables were handled in the analyses. If applicable, describe which groupings were chosen and why	Methods (Potential explanatory variables)
Statistical methods	12	(a) Describe all statistical methods, including those used to control for confounding	Methods (sections on Multiple imputation and Statistical methods)
		(b) Describe any methods used to examine subgroups and interactions	Methods (Statistical methods, second paragraph)
		(c) Explain how missing data were addressed	S2 appendix (Multiple imputation)
		(d) If applicable, explain how loss to follow-up was addressed	S2 appendix (multiple imputation)

Continued on next page...

Strobe checklist (continued)

	**Item No**	**Recommendation**	**Section (notes)**
		(e) Describe any sensitivity analyses	Methods (Statistical methods, second paragraph)
**Results**			
Participants	13	(a) Report numbers of individuals at each stage of study—eg numbers potentially eligible, examined for eligibility, confirmed eligible, included in the study, completing follow-up, and analysed	Results (paragraph 1) and Figure 2.
		(b) Give reasons for non-participation at each stage	Figure 2
		(c) Consider use of a flow diagram	Figure 2
Descriptive data	14	(a) Give characteristics of study participants (eg demographic, clinical, social) and information on exposures and potential confounders	Results (paragraph 2 to 4), and tables 1 and 2
		(b) Indicate number of participants with missing data for each variable of interest	Results (first paragraph of "Sensorimotor outcome at two years of age" section) and tables 1 to 4
		(c) Summarise follow-up time (eg, average and total amount)	N/A
Outcome data	15	Report numbers of outcome events or summary measures over time	Results (first paragraph of "Sensorimotor outcome at two years of age" section) and table 3
Main results	16	(a) Give unadjusted estimates and, if applicable, confounder-adjusted estimates and their precision (eg, 95% confidence interval). Make clear which confounders were adjusted for and why they were included	Confounders are presented in the Methods (section "Statistical methods", paragraph 1). Unadjusted and adjusted estimates are presented in the Results (sections "Sensorimotor outcome at two years of age" and "Morbidity-free survival") as well as tables 4 and 5.
		(b) Report category boundaries when continuous variables were categorized	Methods ("Potential explanatory variables" section) and tables 1 and 2

Continued on next page...

Strobe checklist (continued)

	Item No	Recommendation	Section (notes)
		(c) If relevant, consider translating estimates of relative risk into absolute risk for a meaningful time period	N/A
Other analyses	17	Report other analyses done — eg analyses of subgroups and interactions, and sensitivity analyses	Results (“Sensitivity analyses” section and S3 appendix, tables 1 to 5)
**Discussion**			
Key results	18	Summarise key results with reference to study objectives	Discussion (paragraph 1)
Limitations	19	Discuss limitations of the study, taking into account sources of potential bias or imprecision. Discuss both direction and magnitude of any potential bias	Discussion (“Strengths and limitations” section)
Interpretation	20	Give a cautious overall interpretation of results considering objectives, limitations, multiplicity of analyses, results from similar studies, and other relevant evidence	Conclusion
Generalisability	21	Discuss the generalisability (external validity) of the study results	Discussion (“Study findings in context”, paragraphs 7 to 9)
**Other information**			
Funding	22	Give the source of funding and the role of the funders for the present study and, if applicable, for the original study on which the present article is based	Acknowledgements section (second paragraph)

a Give information separately for exposed and unexposed groups.

Note: An Explanation and Elaboration article discusses each checklist item and gives methodological background and published examples of transparent reporting. The STROBE checklist is best used in conjunction with this article (freely available on the Web sites of PLoS Medicine at http://www.plosmedicine.org/, Annals of Internal Medicine at http://www.annals.org/, and Epidemiology at http://www.epidem.com/). Information on the STROBE Initiative is available at http://www.strobe-statement.org.

Author details

[1]INSERM UMR 1153, Obstetrical, Perinatal and Pediatric Epidemiology Research Team (EPOPé), Centre for Epidemiology and Statistics Sorbonne Paris Cité, DHU Risks in Pregnancy, Paris Descartes University, Hôpital Tenon, Rue de la Chine, 75020 Paris, France. [2]Institute for Womens' Health, UCL, 74 Huntley Street, WC1E 6AU, London, UK. [3]SAMU 93 - SMUR Pédiatrique, CHI André Gregoire, Groupe Hospitalier Universitaire Paris

Seine-Saint-Denis, Assistance Publique des Hôpitaux de Paris, Montreuil, France. [4]UPMC Université Paris 6, Sorbonne Universités, Paris, France. [5] Service de Néonatologie, Hopital Armand Trousseau, Assistance Publique des Hôpitaux de Paris, Paris, France. [6] Maternité Olympe de Gouges, Centre Hospitalier Regional Universitaire Tours, Tours, France. [7] Université François Rabelais, Tours, France. [8] Service de Médecine Néonatale, Centre Hospitalier Intercommunal de Creteil, Clinical Research Center CHI Créteil, Créteil, France. [9] Maternité Port-Royal, University Paris-Descartes, Hôpitaux Universitaires Paris Centre, Assistance Publique des Hôpitaux de Paris, Paris, France. [10] URC CIC P1419, DHU Risk in Pregnancy, Cochin Hotel Dieu, Assistance Publique des Hôpitaux de Paris, Paris, France.

References

1. Vandenbroucke, J.P., von Elm, E., Altman, D.G., Gotzsche, P.C., Mulrow, C.D., Pocock, S.J., Poole, C., Schlesselman, J.J., Egger, M.: Strengthening the reporting of observational studies in epidemiology (strobe): explanation and elaboration. PLoS Medicine **4**(10), 297 (2007). doi:10.1371/journal.pmed.0040297
